# Natural Carotenoids: Recent Advances on Separation from Microbial Biomass and Methods of Analysis

**DOI:** 10.3390/antiox12051030

**Published:** 2023-04-29

**Authors:** Harris Papapostolou, Vasiliki Kachrimanidou, Maria Alexandri, Stavros Plessas, Aikaterini Papadaki, Nikolaos Kopsahelis

**Affiliations:** 1 Department of Food Science and Technology, Ionian University, 28100 Argostoli, Greece; 2 Laboratory of Food Processing, Faculty of Agriculture Development, Democritus University of Thrace, 68200 Orestiada, Greece

**Keywords:** natural carotenoids, colorants, separation of carotenoids, analysis of carotenoids, biotechnological production

## Abstract

Biotechnologically produced carotenoids occupy an important place in the scientific research. Owing to their role as natural pigments and their high antioxidant properties, microbial carotenoids have been proposed as alternatives to their synthetic counterparts. To this end, many studies are focusing on their efficient and sustainable production from renewable substrates. Besides the development of an efficient upstream process, their separation and purification as well as their analysis from the microbial biomass confers another important aspect. Currently, the use of organic solvents constitutes the main extraction process; however, environmental concerns along with potential toxicity towards human health necessitate the employment of “greener” techniques. Hence, many research groups are focusing on applying emerging technologies such as ultrasounds, microwaves, ionic liquids or eutectic solvents for the separation of carotenoids from microbial cells. This review aims to summarize the progress on both the biotechnological production of carotenoids and the methods for their effective extraction. In the framework of circular economy and sustainability, the focus is given on green recovery methods targeting high-value applications such as novel functional foods and pharmaceuticals. Finally, methods for carotenoids identification and quantification are also discussed in order to create a roadmap for successful carotenoids analysis.

## 1. Introduction

Around the beginning of 19th century—where the phrase “You are what you eat” originates—humankind started to realize that human health is unequivocally interlinked with our diet. The flourishing concern regarding environmental pollution is concurrent with an increasing awareness towards safer, healthier and more functional foods. Moreover, based on recent studies about the controversial health effects of synthetic food additives, the limitation and substitution of synthetic compounds with natural products is constantly expanding.

Among food additives, carotenoids occupy a considerable market share, as according to recent market reports, their market size is projected to increase from USD 1.5 billion in 2019 to USD 2.0 billion in 2026 with an annual growth rate of 4.2% [1]. The importance of carotenoids as food additives is pivotal, not only as natural colorants, but also as health promoters due to their antioxidant capacity and their activity as precursors of vitamin A [2]. Vitamin A is essential for the proper function of the eyes and brain, as well as reproduction and other vital activities in the human body; thus, the significance of carotenoids in human nutrition is an indisputable fact. Numerous studies corroborate the multiple positive health effects that a diet rich in carotenoids could offer, from prevention against different types of cancer to protection against depression [3,4]. In this framework, the Food and Agricultural Organization (FAO) and World Health Organization (WHO) have already included several carotenoids in the Codex Alimentarius as safe additives permitted for use in foods. 

Likewise, studies on consumers’ behavior have indicated that food coloration confers a critical parameter associated with food choices. On top of that, the consumption of artificial food colorants has been associated with behavioral disorders in children (the “Southampton study”) [5]. These facts have actually intensified the interest of food industries towards natural colorants. Even though legislation is quite unclear in distinguishing “natural” and “artificial” food colorants, both in the US and the EU, increasing consumer awareness for healthier foods has shifted the scientific pursuit towards pigment-producing microorganisms [5,6]. 

Natural carotenoids can be obtained either by extraction from plants or via microbial production. The implementation of microbial entities to generate carotenoids has been in the spotlight of scientific interest in the last few decades, considering the numerous advantages that biotechnological production exhibits over plant extraction. For instance, biotechnological synthesis of carotenoids suggests an easier scale-up and an enhanced process feasibility, while the utilization of low-cost materials as bioconversion feedstock further minimizes the cost [7]. In fact, the latter merits a significant share of the recently published research dealing with the biotechnological production of carotenoids from algae, bacteria and fungi, focusing on process optimization or even the study of new carotenoid-producing microbial strains. 

On the other hand, exploiting such technology on an industrial scale should require the development of a methodology for the extraction, separation and purification of carotenoids as well as reliable analytical processes. A conceptual approach would consolidate the downstream of carotenoids, the structure identification and the purity determination on-site in a cost-efficient process. Hence, apart from the competent production of carotenoids with microorganisms, an important research aspect should be also devoted to the separation of carotenoids from microbial biomass and their analysis. Additionally, the potential use of carotenoids as food additives or in pharmaceuticals is in line with demands for the use of safer and non-toxic techniques. In this context, the introduction of new instruments and greener technologies has prompted swift development. 

The scope of this article is to elaborate an overview on state-of-the-art developments on carotenoids extraction and purification, encompassing also their biotechnological production. Furthermore, based on recent publications of the open literature, our work emphasizes the conclusive methodology illustration for the accurate and reliable identification and quantification of microbially produced carotenoids.

## 2. Natural Carotenoids

### 2.1. Types and Chemistry of Carotenoids

**Figure 1:**
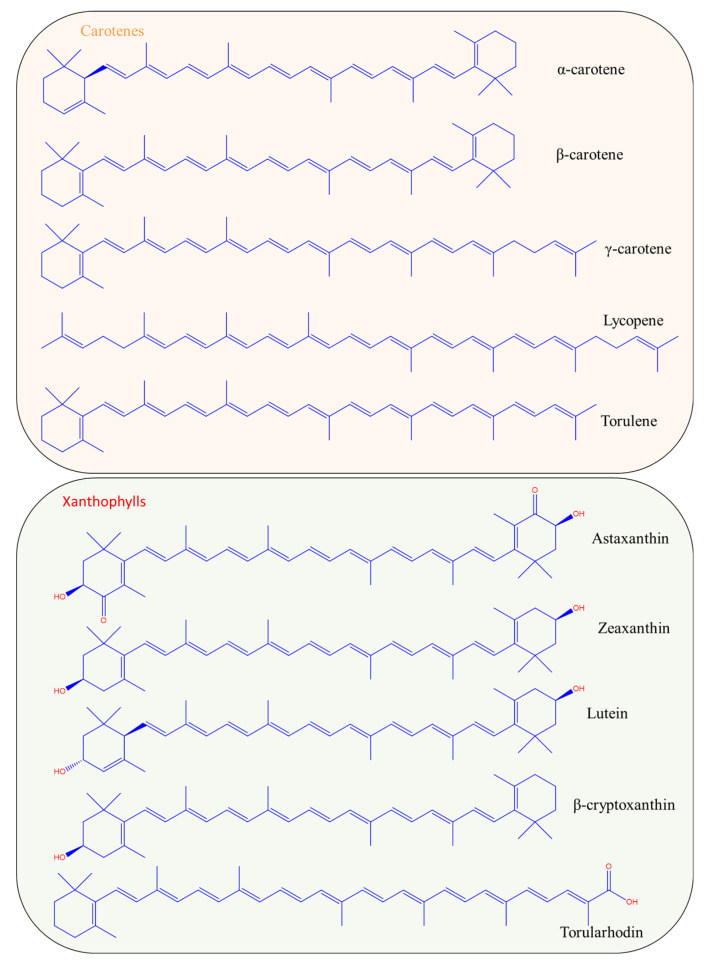
Chemical structures of the most important carotenoids produced biotechnologically.

Carotenoids are chemical compounds belonging to the group of terpenoids. Terpenoids are also called isoprenoids because their structure consists of eight units of isoprene (C_5_H_8_). There are two categories of carotenoids, both with biotechnological interest; the carotenes consist of hydrocarbons, and the xanthophylls with their oxygenated derivatives [8]. Figure 1 presents the chemical structures of the most common carotenes and xanthophylls produced by microorganisms. Carotenes include carotenoids such as *α*-, *β*- and *γ*-carotene, lycopene, torulene, neurosporene and others. The group of xanthophylls includes astaxanthin, zeaxanthin, lutein, torularhodin, canthaxanthin, violaxanthin and others. 

Natural carotenoids are biosynthesized via two major pathways, namely the 2-C-methyl-D-erythritol 4-phosphate (MEP) and the mevalonate (MVA) pathways. The two main terpenoid precursors generated from these pathways are dimethylallyl diphosphate (DMAPP) and isopentenyl diphosphate (IPP), respectively. Geranylgeranyl diphosphate (GGPP) is then generated from IPP isomerization, carried out by the addition of three IPP molecules to DMAPP, which is subsequently condensed to phytoene [9,10]. Finally, phytoene, which is the first carotenoid synthesized from the pathways, is desaturated and isomerized to lycopene [10,11]. The structure of all carotenoids can be derived from the structure of acyclic C_40_H_56_ (corresponds to the structure of lycopene) via hydrogenation, dehydrogenation, cyclization or oxidation [8]. The main characteristic of the carotenoid structure is the long hydrocarbon chain, consisting of conjugated double bonds. The delocalized π electrons from the conjugated double bonds system are the reason that carotenoids exhibit high antioxidant properties. In β-carotene, both ends are cyclized, while lycopene is characterized by two acyclic parts in its structure. The number of conjugated double bonds is also linked to the color of the carotenoid [12]. Molecules with a high number of conjugated double bonds absorb at higher wavelengths, resulting in a yellow-red color. A characteristic example is the formation of lycopene during the maturation of tomatoes from phytoene by phytoene desaturase. The conjugated bonds in the carotenoids’ structure have the ability to absorb visible light and are thus responsible for the molecule’s color. Phytoene is a colorless compound with three conjugated double bonds that is converted to phytofuene (pale yellow) with five conjugated double bonds, *ζ*-carotene (yellow) with seven conjugated double bonds, neurosporene (orange) with nine conjugated double bonds and finally to lycopene (red) with eleven conjugated double bonds [13]. 

The number of conjugated bonds in the molecule determines the color and the absorbance maxima. For instance, lycopene is an acyclic molecule of red color and because of the 11 conjugated double bonds, its maximum absorbance is located at 444, 470 and 502 nm. On the other hand, α-carotene, with 10 conjugated double bonds (one of them in the cyclic part), absorbs at 422, 445 and 473 nm, demonstrating an orange color [12].

**Figure 2:**
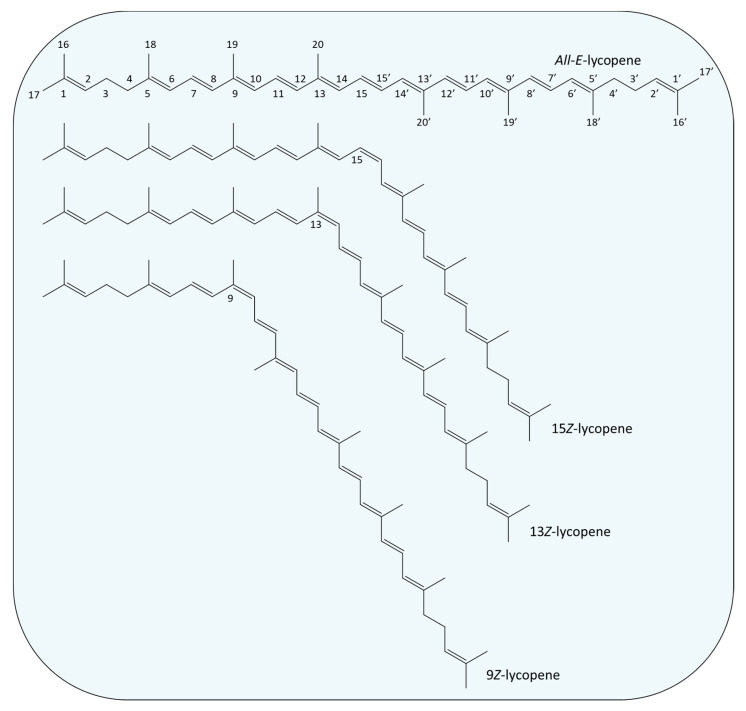
Examples of chemical structures of *trans* (*All*-*E*) and *cis* isomers (*15Z-, 13Z*- and *9Z*-) of lycopene.

The position of the side groups linked to the atoms where the double bonds are located divides carotenoids into *trans* or *cis* isomers. *Trans* carotenoids are also characterized as *all-E* and *cis* carotenoids as *Z* [14]. The *all-E* characterization refers to a structure where all double bonds are in *trans* formation, while the *Z* one differs depending on the double bond that is found in *cis* formation. Figure 2 illustrates the *all-E* isomer (*trans*) of lycopene and one of its *cis* isomers. Carotenoids occur predominantly in their *all-E* form; however the extent of antioxidant activities and bioavailability in the human body is highly dependent on the type of carotenoid under investigation [15]. Many studies evidence that *Z*-isomers (especially in the case of lycopene and astaxanthin) exhibit higher scavenging activities than their *trans* counterparts, while also presenting greater bioavailability [15,16,17]. Likewise, regarding their abundance, there is a wide distribution of carotenoid isomers in nature. For example, β-carotene is commonly found in *all-E*, as well as in its *9Z*- and *13Z*- isomers; lycopene also occurs as *5Z*-, *9Z*-, *13Z*-, *15Z*- and *di-Z*-, while *9Z*-lutein and *13Z*-lutein have been identified in tomatoes [16].

Chirality is another characteristic of most carotenoids due to the presence of chiral centers in their molecules. Astaxanthin and zeaxanthin are characteristic examples of chiral carotenoids, both with two chiral centers in 3 and 3′ carbon due to the presence of hydroxyl groups. These carotenoids have two enantiomers—the 3R,3′R and the 3S,3′S—and an optical inactive meso form of 3R,3′S [18]. 

### 2.2. Sources of Natural Carotenoids

Carotenoids are naturally occurring in plants, photosynthetic bacteria and algae but also in some heterotrophic bacteria and fungi [13,14]. Animals and humans obtain carotenoids from food, as they are unable to synthesize them. Nevertheless, the obtained carotenoids can be modified with metabolic reactions [13] for the synthesis of other carotenoids or their derivatives.

#### 2.2.1. Plants

Plants constitute the prevalent source of natural carotenoids, as a wide spectrum of these compounds is found in fruits, flowers and vegetables but also in other plant tissues such as leaves, roots and seeds [19]. *β*-carotene is the predominant carotenoid extracted from plants, with carrots, spinach, tomatoes, sweet potatoes, broccoli and lettuce to be the richest sources. According to Stephen et al. [20], *α*- and *β*-carotene are the signature carotenoids of carrot, lycopene is for tomatoes, watermelon and papaya, lutein is for watercress and spinach and yellow bell peppers are rich in violaxanthin. 

The utilization of waste fruits and vegetables for the sustainable recovery of carotenoids has been already demonstrated [21]. Another interesting approach has been recently introduced by Metličar et al. [22,23], proposing the exploitation of invasive alien plant species such as Japanese knotweed and Bohemian knotweed to extract carotenoids and xanthophylls. 

#### 2.2.2. Microorganisms

Besides plants, several microbial species, belonging to algae, bacteria and fungi, are able to accumulate intracellularly different types of carotenoids as metabolic products. Carotenoids production in non-photosynthetic microorganisms is connected to an evolutionary response to photo-oxidative damage caused by light and oxygen-rich habitats [24]. In the last few decades, many research groups have prioritized the investigation on the biotechnological production of carotenoids. It is only indicative that from the 750 naturally occurring carotenoids, more than 600 can derive from microorganisms [25,26].

Plant-derived pigments display difficulties with respect to characterization and standardization due to the effect of cultivation and climate conditions. Another important drawback lies in the stability and functionality of these pigments, particularly regarding exposure at high temperatures, pH variations and light [27]. Microorganisms evidence a prominent scientific potential due to the variation of their pigment color, the chemical profile of the pigment, the independence of seasonal restrictions and climate conditions, along with the capacity to scale up. In the framework of sustainability and circular economy, it is nowadays imperative to explore alternative sources of food additives that will not compete with food and feed and, in parallel, will respect the environment. Microbial carotenoids coincide entirely with the above mentioned purposes. The efficient productivities and high yields, complete process control and standardization of the product quality confer advantages of microbial carotenoids’ synthesis, directly related to the potential for large-scale production. Moreover, the utilization of low-cost and renewable resources (such as agro-industrial wastes and byproducts) as substrates for microbial growth could further mitigate the overall cost of production [28].

### 2.3. Biotechnological Production of Carotenoids

Table 1 refers to indicative research of the last five years on the biotechnological production of carotenoids. In fact, algae and fungi are the most popular choices among the most studied microbial strains for carotenoid production such as *β*-carotene, astaxanthin, torulene, zeaxanthin, torularhodin and lutein. 

Microalgae are widely recognized as sources of diversified bioactive compounds such as pigments, phenolic compounds, fatty acids, proteins and vitamins, among others [24]. The microalgae *Haematococcus pluvialis* and *Dunaliella salina* have been extensively studied for astaxanthin and *β*-carotene production, respectively [24,29,30,31]. Spirulina is richer in *β*-carotene even compared to carrots [27]. *Chlorella* and *Scenedesmus* are also significant carotenoid producers, mainly of lutein [32,33]. The limitation of large-scale carotenoid production using microalgae lies in the high production costs and the high land requirements [28,34]. Bacteria and fungi introduce a prevalent advantage to this angle, as proper strain selection, substrate and fermentation/bioreactor design can lead to high product yields and productivities in completely controlled processes.

Yeasts have emerged as robust carotenoid producers, with the strains *Rhodotorula* sp. and *Phaffia rhodozyma* occupying most of the recently published works. *Rhodotorula* sp. together with the genera *Rhodosporidium*, *Sporidiobolus* and *Sporobolomyces* belong to a category known as “red yeasts”, describing their ability to intracellularly accumulate carotenoids [35]. Another important attribute of yeasts is their capability to synthesize carotenoids via the valorization of low-cost substrates as growth substrates, including cheese whey [28], molasses [36] and raw glycerol [37]. Red yeasts have been generally reported to produce mixtures of carotenoids, mainly *β*-carotene, *γ*-carotene, lycopene, torulene and torularhodin [28,35]. *P. rhodozyma*, a basidiomycetous yeast, is a well-known producer of astaxanthin and *β*-carotene [27,38]. The fungus *Blakeslea trispora* is industrially employed for *β*-carotene and lycopene production and its use as a food additive has already been approved [39,40]. 

Likewise, recent studies have been undertaken using some Archaea for carotenoid synthesis. Many Haloarchaea have developed the ability to accumulate pigments as a response to stress factors. To this end, Giani et al. [41] investigated the potential of the strain *Haloferax mediterranei* to synthesize carotenoids, and especially the C_50_ carotenoid bacterioruberin, when subjected to various concentrations of H_2_O_2_. Similarly Lizama et al. [42] explored different Haloarchaea strains, namely *Halorubrum tebenquichense* and *Haloarcula* sp., for their carotenoid profile and antioxidant capacity. 

Finally, cyanobacteria are also able to produce carotenoids, still in lower amounts compared to other pigments such as phycocyanin [43]. Pagels et al. [43] recently reviewed the potential of these microorganisms, reporting the strains *Cyanobium* sp., *Arthrospira platensis*, *Trichodesmium* sp. and *Lyngbya* sp. as some characteristic examples of carotenoid-producing strains, also exhibiting antioxidant activities. The main carotenoids synthesized by these strains were *β*-carotene and zeaxanthin.

**Table 1:** Recent developments on natural carotenoids produced by microorganisms.

Main Carotenoid Produced	Microbial Strain	Ref
*α*-carotene	*Rhodotorula mucilaginosa*	[44]
*β*-carotene	*Rhodotorula glutinis CCT-2186*	[45]
	*Xanthophyllomyces dendrorhous*	[46]
	*Phaffia rhodozyma*	[47]
	*Rhodotorula mucilaginosa*	[44]
	*Blakeslea trispora*	[48]
	*Dunaliella salina CCAP 19/41*	[49]
	*Rhodosporidium kratochvilovae Y-42* and *Y-43*	[28]
*γ*-carotene	*Rhodotorula mucilaginosa* *Blakeslea trispora*	[44] [48]
Lycopene	*Blakeslea trispora*	[48]
Torulene	*Rhodotorula glutinis CCT-2186*	[45]
	*Rhodotorula mucilaginosa*	[44]
Astaxanthin	*Xanthophyllomyces dendrorhous*	[46,50,51]
	*Phaffia rhodozyma*	[47]
Zeaxanthin	*Flavobacterium* sp. P8	[52]
	*Synechococcus* sp. PCC7002, *Synechocystis* sp. PCC6803 and *Rhodosorus* sp.	[53]
Lutein	*Asterarcys quadricellulare PUMCC 5.1.1*	[54]
	*Auxenochlorella spp. LEU27*	[55]
	*Chlorella minutissima*	[56]
	*Chlorella pyrenoidosa*	[57]
	*Chlorella sorokiniana AK-1*	[58]
	*Chlorella sorokiniana FZU60*	[59,60]
	*Chlorella sorokiniana MB-1-M12*	[61,62,63]
	*Chlorella sorokiniana MUM002*	[64]
	*Chlorella saccharophila UTEX247*	[65]
	*Chlorella sp. GY-H4*	[66]
	*Chlorella vulgaris*	[67]
	*Tetraselmis sp. CTP4*	[68]
	*Scenedesmus sp.*	[69]
Torularhodin	*Sporobolomyces ruberrimus*	[70]
	*Rhodotorula glutinis CCT-2186*	[45]
	*Rhodotorula mucilaginosa*	[44]

## 3. Extraction of Microbial Carotenoids

There are two steps employed in the extraction of carotenoids from the microbial biomass, namely, the disruption of the microbial cell membrane and the extraction of the carotenoids. Carotenoids are formulated intracellularly; thus, it is important to disrupt the microbial cells prior to extraction. A wide range of methods targeting cell disruption are found in the literature; the selection of the appropriate method closely depends on the type of microorganism employed in carotenoids production, along with the end application designated for the extract. In general, bacterial cells are much easier to be ruptured, in comparison to yeast or microalga cells that possess a more rigid and complex cell wall [7]. Cell disruption with DMSO is the most traditional and efficient method still applied by many researchers. Mechanical disruption is also commonly used, depending on the strain utilized as a biocatalyst.

Carotenoids can be easily degraded by exposure to light, high temperatures or solvents. The selection of the appropriate steps and procedures is crucial for maintaining their stability [71]. The presence of water in microbial biomass is considered unfavorable for carotenoids’ extraction due to their hydrophobicity. To this end, biomass lyophilization is often carried out; however, this process extends both time and costs [71]. The efficiency of the extraction process in wet biomass has been investigated in certain yeast cells, where the results were comparable to dried or freeze-dried biomass [28]. Regardless of the extraction process, carotenoid samples should be protected from UV light to avoid *trans–cis* photoisomerization [71]. Flushing the samples with nitrogen constitutes a typical technique to eliminate oxygen.

Dimethyl sulfoxide (DMSO) is typically employed for the recovery of carotenoids from cell biomass owing to its ability to dissolve a wide array of analytes. DMSO is a polar aprotic solvent that presents high affinity for carotenoids without either reacting or degrading them during the downstream process. 

The next step involves the recovery of carotenoids from other metabolites and cell slurry. Solvent extraction offers high extraction yields; however, the toxic effects demonstrated by the prevalently used chemicals towards both human health and the environment have urged the scientific community to search for greener alternatives [7]. To this end, several methods have been developed, such as ultrasound-assisted extraction (UAE), microwave-assisted extraction (MAE), enzyme-assisted extraction (EAE) and ionic liquids or supercritical fluid extraction.

Table 2 displays the recent developments not only on extraction but also on carotenoid analysis.

### 3.1. Solvent Extraction

Although solvent extraction is a conventional method for carotenoids recovery from microbial biomass, it is unequivocally the most universal. Extensive research on the application of solvent extraction for the recovery of various compounds has entailed important progress regarding process optimization and solvent selection. 

Gong et al. [73] extracted carotenoids from recombinant *E. coli* cells using acetone as an extracting solvent. Initially, the cells were separated from the fermentation broth by centrifugation, followed by resuspension in acetone and incubation for 15 min at 55 °C in the dark. Finally, the samples were centrifuged again and carotenoids were collected with the supernatant. Huang et al. [74] also used acetone in order to extract carotenoids from algal cells. In this case the cells were lyophilized, the carotenoids were extracted from the cells directly and then the extract was filtered prior to analysis. 

Vila et al. [52] extracted carotenoids from lyophilized cells of *Flavobacterium* sp. P8 strain using methanol. After the extraction, the solvent was removed with evaporation and the carotenoids were dissolved in acetone for further analysis. 

Sereti et al. [28] utilized DMSO as a cell-disrupting agent in lyophilized cells of *R. kratochvilovae* Y-42 and Y-43. The cells were initially separated from the fermentation broth via centrifugation, while the treatment with DMSO was carried out for 1 h in a water bath at 40 °C. The second step involved the addition of acetone for cell pellet precipitation, and the process was repeated until the cells were fully decolorized. Finally, carotenoids were obtained from the pigment-rich liquid phase by solvent extraction with petroleum ether.

In a recent publication, Bourdon et al. [53] reported the combination of solvent extraction and mechanical cell disruption for carotenoids recovery. In this case, the microalgal cells were harvested by the cultivation medium, washed with deionized water and lyophilized. The dry cell mass was disrupted using zirconium beads, and the carotenoids were extracted with methanol. A similar process was also followed by Byrtusova et al. [78] to recover carotenoids synthesized by the yeast *R. kratochviloave* CGY 20-2-26. More specifically, cell lysis was performed by vigorous vortexing with acid-washed glass beads and methanol. Carotenoids extraction was subsequently conducted using chloroform. 

The use of different solvent combinations to obtain carotenoids from the yeast *Sporobolomyces ruberrimus* has been recently investigated [70]. In this case, the disruption of microbial cells was undertaken using glass beads, whereas several combinations of the organic solvents hexane, acetone, petroleum ether and ethyl ether were employed in the extraction step. The highest amount of carotenoids from *S. ruberrimus* was achieved using the solvent mixture of acetone: hexane (9:1 *v*/*v*) corresponding to a value of 221.88 μg carotenoids/g of cells. 

Carotenoids extraction using natural monoterpenes has been recently shown as an eco-friendly alternative to conventional solvents [23,79]. In the work of Boukroufa et al. [79], D-limonene recovered from citrus wastes was employed as a green extraction solvent for carotenoids recovery from the same waste stream. The extraction of xanthophylls and lutein from avocado peels and green leaves of Japanese knotweed was achieved using β-pinene, which was also utilized as a bio-solvent for the synthesis of xanthophyll esters [23]. Even though these solvents have not been tested yet on microbial biomass, they could be considered as an interesting green approach. 

### 3.2. Green Technologies for the Separation of Carotenoids

The majority of publications corroborate the efficiency of DMSO as a first step for carotenoids extraction from microbial cells; still, the toxicity of its derivatives poses a threat towards human health. Hence, green and safer alternatives need to be investigated, particularly when the end product is designated for high added value formulation. Therefore, the implementation of green technologies to extract bioactive compounds including carotenoids demonstrates state-of-the-art development, notably when the production of food additives is co-opted. 

**Figure 3:**
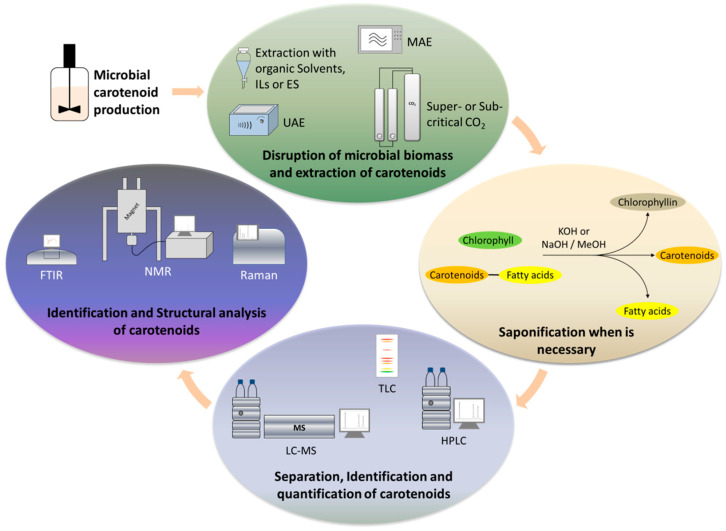
Main stages of extraction and analysis of microbially produced carotenoids.

The whole concept is based on the use of technologies that can minimize or replace the use of toxic solvents, still leading to similar or higher extraction yields. Such technologies include the use of ultrasounds, microwaves, enzymes, supercritical fluids and ionic liquids or deep eutectic solvents (DES) (Figure 3). 

#### 3.2.1. Ultrasound-Assisted Extraction (UAE)

Solvent extraction assisted by ultrasounds is one of the most commonly applied, non-conventional technologies to extract bioactive compounds such as carotenoids. Advantages of this method include lower extraction times, the use of low temperatures and low energy and solvent demands [6,80]. In this process, the application of ultrasounds targets the disruption of the microbial cell wall in order to maximize carotenoids’ extraction from the solvent. This method is based on the formation of bubbles in the targeted solution, followed by changes in the pressure caused by the ultrasounds (cavitation phenomenon). Subsequently, the bubbles burst and generate shock waves that rupture the cell membrane [81]. 

Fidan and Zhan [72] recovered carotenoids from wild type and genetically engineered bacterial strains using sonication for cell disruption, in combination with methanol. Finally, the solvent was removed and the carotenoids were redissolved in a DMSO–methanol mixture for HPLC analysis. 

A similar process was employed by Sarnaik et al. [82] for the extraction of zeaxanthin from three different microalgae and three cyanobacteria. After the separation of the microbial biomass from the liquid by centrifugation, the cells were suspended in absolute methanol and the extraction of the pigments was assisted by sonication. 

In the work of Park et al. [83], astaxanthin was recovered from metabolic engineered *E. coli* cells via solvent extraction with acetone assisted by ultrasounds. Following sonication, the disrupted microbial cells were vortexed and the extract was separated by centrifugation. 

As in any other process, there are some impediments to the widespread use of ultrasounds for carotenoids extraction. Another review [71] highlighted that ultrasonic power, intensity, temperature and sample-to-solvent ratio constitute crucial factors to be optimized for a successful UAE. Furthermore, according to the same publication, the use of ultrasounds, with intensity higher than the optimum value, induces the formation of free radicals such as ^●^OH and ^●^H that can affect the structure and antioxidant activity of phenolics and carotenoids. It is evident that the selection of the appropriate UAE parameters are of paramount importance to avoid low recoveries or even degradation of carotenoids [84].

#### 3.2.2. Microwave-Assisted Extraction (MAE)

The extraction of carotenoids from microorganisms with the assistance of microwaves represents another reported green technology. Similar to sonication, this technique exploits the energy of microwave radiation to disrupt the microbial cell wall aiming at a more efficient solvent extraction. During microwave extraction, an elevation of temperature and pressure occurs, leading to the rupture of the cell membrane. Subsequently, carotenoids are released in the solvent, and finally the solvent is diffused in the cell matrix [85]. This method is based on the rapid heating of the intracellular components that creates high pressure on the cell wall [86]. The energy of the microwave irradiation is transferred to the components either by using a polarization dipole or by ionic conductivity [87]. MAE could find application particularly in the case of microalgae, because of the strong complexity and resilient cell wall structure [86]. 

Sarma et al. [88] investigated the extraction of carotenoids from *Chlorella* sp. with microwaves using different extraction solvents. The authors optimized the process using response surface methodology (RSM) for the different extraction systems. Acetone, *n*-hexane, methanol and mixtures of *n*-hexane: acetone (1:1) and n-hexane: ethanol (7:3) were evaluated. The higher extraction yield was achieved using acetone as an extraction solvent resulting in the recovery of 0.063 mg/g of total carotenoids, similar to the predicted value of the model. It is important to mention that a program of 30 s heating and 30 s cooling was applied to minimize carotenoid degradation from overheating. The authors stated that this process promoted efficient cell disruption and consequently higher extraction yields.

Fabrowska et al. [87] compared different extraction methods for the recovery of carotenoids from the freshwater green algae *Cladophora glomerata*, *Cladophora rivularis* and *Ulva flexuosa*. The authors conducted experiments using UAE, MAE, supercritical fluid extraction (SFE) and conventional solvent extraction for the recovery of chlorophylls and carotenoids. Based on the results in all three strains, the utilization of ultrasounds or microwaves suggested the most efficient methods for carotenoids recovery. 

Despite the efficiency of the MAE method, there are still some drawbacks regarding its application on carotenoid extraction. As aforementioned, the basis of the method is the elevation of temperature inside the microbial cells, hence some limitations arise relating to the thermo-sensitivity of carotenoids. Sarkar et al. [86] utilized a high-speed tissue homogenizer for cell disruption and MAE with ethanol as the extraction solvent, aiming to optimize the extraction of carotenoids from *Chlorella thermophile*. A 5% and 25% increase in the extraction efficiency was achieved for wet and dry algae using 4.5 min microwave pretreatment. Extraction efficiency was not affected by the pretreatment time. The authors declared that the impediment of this method could be associated with the degradation of carotenoids due to extensive use of microwaves. 

#### 3.2.3. Enzyme-Assisted Extraction (EAE)

Τhe utilization of hydrolytic enzymes introduces an additional green technique to facilitate carotenoids release from microbial cells. Enzymes have been mainly used for the recovery of plant carotenoids, whereas enzyme utilization for microbial cell lysis has been employed to obtain several biotechnological products, including PHAs [89]. However, only a handful of researchers have applied enzymes in the recovery of microbial carotenoids, probably due to the high costs. In any case, proteases and cellulases or enzyme mixtures are nowadays employed for microbial cell lysis. The commercial enzyme preparations Alcalase^®^ and Viscozyme^®^ were recently evaluated for the rupture of the microalgae *N. oculata* [77]. Alcalase^®^ proved to be more efficient, providing comparable extraction yields to the combination of UAE with ethanol. Glucanex^®^ has also been investigated to disrupt the yeast cells of *P. rhodozyma* [90]. Other enzymes previously tested were lyticase and lipase. Lipase addition aids not only in lipids hydrolysis (carotenoids are often “trapped” in lipid droplets), but also facilitates the subsequent HPLC analysis by avoiding capillary plugging [91]. The combination of EAE with another method—mainly UAE—results in higher extraction yields rather than as a sole cell disruption method.

#### 3.2.4. Supercritical Fluid Extraction (SFE)

Supercritical fluids have lately gained scientific attention owing to their properties and extraction capacities compared to conventional methods. Any compound at temperature and pressure above the critical point exists in both liquid and gas form, exhibiting properties from both phases. For example, a supercritical fluid can diffuse through the pores of a solid like a gas but can also dissolve materials like a liquid [92]. These properties render supercritical fluids suitable as solvents for the extraction of sensitive compounds such as carotenoids. Likewise, the temperature and pressure applied are the most notable parameters and, along with the extraction time and solvent composition, denote the most investigated factors in SFE in the literature. SFE combined with CO_2_ as the extracting solvent is employed in carotenoids recovery, since CO_2_ is considered a GRAS solvent with high selectivity towards non-polar, lipophilic compounds [93].

The extraction of lutein using SFE has been recently assessed [94]. In this research, authors conducted experiments to optimize the extraction of lutein from the microalgae *Scenedesmus almeriensis* using supercritical CO_2_ as the extraction solvent. The highest recovery of lutein, corresponding to 97.6%, was achieved with a CO_2_ flow rate of 14.48 g/min at 65 °C and pressure of 55 MPa. Under these conditions, a 17% lipid recovery and 15% fatty acid recovery from *S. almeriensis* biomass was also accomplished. The extraction of astaxanthin and lutein from the microalgae *Haematococcus pluvialis* using SFE has been also reported [95]. In this case, CO_2_ was used as the extraction solvent with and without ethanol as a co-solvent. More specifically, different temperatures and pressures were tested when the flow rates of CO_2_ and ethanol were kept constant at 3.62 g/min and 1 mL/min, respectively. The optimum conditions for astaxanthin and lutein extraction were 65 °C and 550 bar pressure, leading to 92% and 93% recovery, respectively. These things considered, the use of ethanol as a co-solvent seemed to be more effective in the case of lutein than astaxanthin.

Morcelli et al. [96] studied the extraction of carotenoids from *Chlorella sorokiniana* biomass using SFE with CO_2_ and ethanol mixture. Experimental design and statistical analysis were used to evaluate the solvent composition on the extraction of chlorophylls and carotenoids. Their results corroborate that the use of ethanol displayed an important effect on the extraction of chlorophylls, also acting as a polarity modifier aiding in the extraction of carotenoids. 

#### 3.2.5. Ionic Liquids and Deep Eutectic Solvents

Ionic liquids (ILs) are salts with a low melting point, and deep eutectic solvents (DESs) are binary or ternary mixtures of compounds with much lower melting points than the corresponding individual compounds. Because of these similar properties, both ILs and DESs can be applied as extracting solvents of bioactive compounds. By definition, ILs are fluids consisting solely of ions and have a melting point lower than 100 °C. Although ILs and DESs belong to the same category with similar physical properties, they present different chemical properties. ILs consist of one type of anion and cation, whereas DESs can contain more than one anionic and/or cationic species [97]. In the last few years, ILs and DESs have been extensively studied as extraction solvents for numerous compounds including carotenoids. 

Mussagy et al. [45] studied the efficiency of 12 different protic ionic liquids (PILs) for the disruption of cells of *Rhodotorula glutinis* and the extraction of *β*-carotene, torulene and torularhodin. The authors used DMSO for cell disruption as a control and according to the results the use of [Hex]^−^ based PILs (Propylammonium hexanoate, 3-Dimethylamino-1- propylammonium hexanoate and 3-Diethylamino—propylammonium hexanoate) led to the highest recovery of *β*-carotene and torularhodin, while in some cases extraction yields were six times higher than the ones obtained with DMSO.

Paliwal et al. [65] tested four different ionic liquids for the extraction of lutein from the algal *Chlorella saccharophila* without any cell disruption prior to the extraction. Similarly, in another research study [64], alkyl carbamate ILs were evaluated for the extraction of lutein from the algal *Chlorella sorokiniana*. In an attempt to increase cell permeability and subsequently the extraction of carotenoids, the authors tested four different ILs, namely, dimethylammonium dimethylcarbamate, dipropylammonium dipropylcarbamate, diallylammonium diallylcarbamate and dibutylammonium dibutylcarbamate. The maximum lutein amount, more than 971 mg/g, was extracted using a solvent combination of dipropylammonium dipropylcarbamate and methanol (8:2 *v*/*v*) at the optimum conditions of 45 min extraction time and at room temperature. 

One of the most important categories of ionic liquids are eutectic solvents (ESs). ESs have in fact been in the research spotlight during the past few years. In the context of substituting the conventional organic solvents for green ones, Mussagy et al. [47] investigated the extraction of astaxanthin and *β*-carotene from the yeast *Phaffia rhodozyma* using different ILs and ESs as extracting solvents. In their work, the ILs choline lactate ([Ch][Lac]) and choline butanoate ([Ch][But]), as well as the ESs choline chloride + lactic acid ([Ch]Cl: Lac) and choline chloride + butyric acid ([Ch]Cl: But) (in molar ratios of 1:1 and 1:2), were evaluated as extraction solvents. According to the results, higher recovery yields for astaxanthin and *β*-carotene were achieved using [Ch]Cl: But in comparison with the extraction with DMSO. Furthermore, the authors optimized the extraction with the selected solvent by testing different molar ratios of the cation and anion of the ES, different water content of the ES solution and different solid–liquid ratio (wet cells–solvent). The results indicated that the optimum combination of hydrogen bond acceptor: hydrogen bond donor was 1:5, and with this ratio, the highest yield of astaxanthin and *β*-carotene extraction was achieved with less than 20% water content and 0.2 g/mL wet cell to solvent ratio. Gkioni et al. [77] evaluated different extraction and separation methods to recover carotenoids-rich extracts from the microalgae *Nannochloropsis oculata*. A combination of UAE and ES proved promising for the extraction of carotenoids from the microalgae. The ES used was a mixture of betaine: 1,2-propanediol.

## 4. Analysis of Carotenoids

The successful identification and quantification of the produced carotenoids confers a step of paramount importance for the subsequent application as food additives. To this end, different analytical methods have been investigated and presented in the recent literature for the analysis of carotenoids in the extracts. Among these methods, liquid chromatography, thin-layer chromatography and mass spectrometry are the most popular and effective methods for identification, followed by nuclear magnetic resonance (NMR), infrared (IR) and Raman spectrometry for structural analysis. This section discusses the implementation of conventional and modern analytical tools as well as some pretreatment steps that could be necessary for sample preparation (e.g., saponification).

### 4.1. Sample Pretreatment before Analysis—Saponification

While carotenes occur in their free form, xanthophylls occasionally exist in their esterified form with fatty acids, which stabilizes their molecules [98]. Saponification is usually applied prior to the analysis of carotenoids not only to remove chlorophylls (in plant- or algae-derived samples) and lipids, but also to hydrolyze the xanthophyll-fatty-acid esters. The presence of esterified carotenoids during the analysis could lead to false quantitative results when chromatographic methods are employed [98]. 

Saponification can be also applied when we have to determine whether or not the sample contains esterified carotenoids. During the comparison of the chromatogram of a non-saponified sample with the chromatogram of the same sample after saponification, the esterified carotenoids will be absent in the latter; thus, one can easily determine the peaks belonging to the esterified carotenoids [99].

Saponification, also known as alkaline hydrolysis, is the formation of fatty acid salts (soaps) derived from the hydrolysis of their esters with bases such as KOH or NaOH. A typical process involves the dilution of the extract in ethanol, methanol or isopropanol and the addition of concentrated solution of KOH. The reaction takes place in the absence of oxygen and light. During the first two hours of the reaction, chlorophylls are totally converted to water-soluble chlorophyllin, however longer time is required for the carotenoids’ esters to be hydrolyzed [99]. 

Nevertheless, this process should be carried out carefully in order to avoid losses of carotenoids [100]. Hong et al. [101] investigated the effect of different saponification conditions on carotenoids recovery from avocado. According to this research, reaction time and concentration of KOH had an important impact on the extraction yield of carotenoids. The increased KOH concentration and reaction time resulted in enhanced carotenoid recovery, reaching a maximum yield with 15% KOH concentration at 60 min. Higher KOH concentrations led to lower carotenoids recovery as well as higher reaction times. In microbial samples, interferences from chlorophylls are common in the cases in which microalgae are employed. For example, Casella et al. [31] performed saponification with 0.05 M NaOH in methanol for lipids and chlorophylls removal in astaxanthin-rich samples derived from *H. pluvialis* cells. In this case, 20 mg of total astaxanthin/g biomass were recovered.

In general, saponification with KOH in ethanol or methanol can result in carotenoids’ degradation [55]; thus, optimization of the process is usually required, aiming to obtain the optimum results always based on the carotenoids extract to be analyzed.

### 4.2. Thin-Layer Chromatography

Thin-layer chromatography (TLC) is a simple and rapid analytical procedure applied as a first step for the identification of individual carotenoids. Usually, TLC is followed by a more sophisticated and accurate technique such as HPLC or LC-MS for the carotenoids quantification in the sample. 

One of the advantages of TLC is that with a low-cost procedure, pigments can be easily separated for further analysis. Maswanna and Maneeruttanarungroj [102] undertook the production of pigments from *Tetraspora* sp. After the extraction of pigments, the crude extract was dried and redissolved in methanol, and the separation of the pigments was carried out using a TLC plate with silica gel and diethyl ether as the mobile phase. From the TLC analysis, four bands were collected from the plate, and the pigments were extracted with diethyl ether for further analysis. 

TLC analysis can provide a satisfactory separation of carotenoids via the selection of the proper mobile phase system based on the polarity of the analytes. In the recent work of Mishra et al. [103], the production of carotenoids from the bacterial strain *Azospirillum brasilense* was investigated, whereby pigment determination was implemented as a first step via TLC analysis. The authors evaluated different solvent combinations, and the formed bands were significantly more in the case of hexane/acetone (7:3) than in the acetone/chloroform (1:1) mobile phase system. 

TLC analysis can be also assisted by specialized equipment for higher accuracy and reliability. Hynstova et al. [104] used high performance thin-layer chromatography (HPTLC) for the separation and analysis of chlorophylls, carotenoids and pheophytins in commercial dietary supplements containing *Chlorella vulgaris* and *Spirulina platensis*. The apparatus used for the analysis consisted of a TLC plate cutting instrument, a sample applicator, an automatic developing chamber, a TLC scanner and visualizer and the corresponding software. According to this research, the best solvent system for separation of carotenoids, chlorophylls and pheophytins was the combination of petrol medical: isopropanol: water (100:10:0.25, *v*/*v*/*v*). A mobile phase consisting of petroleum ether: cyclohexane: ethyl acetate: acetone: ethanol (60:16:10:10:6, *v*/*v*/*v*/*v*/*v*) was also used for the separation of astaxanthin from chlorophylls. 

Evidently, TLC is a very useful and prominent technique for the separation of carotenoids, given that the only parameter we have to take into account is the polarity of the analytes and the selection of a suitable solvent combination as the mobile phase. 

### 4.3. Liquid Chromatography

Carotenoids have different polarities, mainly depending on the functional groups attached to the ends of the acyclic backbone [18]. The most common technique in carotenoid analysis with liquid chromatography is reversed-phase chromatography. This technique refers to the use of a non-polar compound as a stationary phase and a polar solvent as a mobile phase. The chromatographic columns that are usually employed in carotenoids analysis consist of C18 or a C30 stationary phase. Saini et al. [105] presented an elution sequence of important carotenoids with a C30 chromatographic column based on their polarity. According to this report, neoxanthin and violaxanthin are the most polar molecules followed by lutein, zeaxanthin and cryptoxanthin; finally, α-carotene, *β*-carotene and lycopene are the non-polar ones. This sequence shows that the most polar carotenoids belong to the group of xanthophylls, followed by molecules with cyclic groups such as *α*- and *β*-carotene and finally the acyclic molecule of lycopene. This small but important difference in the polarity of carotenoids demonstrates the principle behind the chromatographic separation and identification.

The separation with both column types is mainly based on the polarity of carotenoids. Nevertheless, in the case of C30, the bounded static phase strongly interacts with the long linear conjugated carotenoids, resulting in a different elution sequence from C18. This can also affect the elution time of geometric isomers, as all-trans isomers strongly bind to C30 static phase because of their linear structure in contrast to all-cis isomers [99]. As a result, C30 chromatographic columns are very effective in separating *cis* and *trans* isomers of carotenoids. To conclude, when a C18 column is selected, the elution sequence can be based only on the polarity of the analyte, whereas in C30 analysis, other parameters, such as the length of the linear part of the molecule, are also involved [99].

On the other hand, the mobile phase varies depending on the sample to be analyzed and the efficiency of the separation. Methanol, water, MTBE, acetonitrile and acetone are often applied as mixtures in isocratic or gradient elution systems in order to create a mobile phase with the proper polarity for the optimum separation of carotenoids (Table 2). As in phenolic compounds’ analysis, the use of a gradient elution system can be equally favorable to analyze carotenoids. Gradient systems can be modified based on the sample to be analyzed in order to maximize separation. 

Lourenço-Lopes et al. [106] proposed an optimized method for HPLC analysis of fucoxanthin, *β*-carotene and chlorophyll produced from nine brown algae. Different combinations of solvents for the mobile phase, different flow rates but also different gradient programs were evaluated, aiming to optimize carotenoid separation. Finally, the best separation of carotenoids and chlorophylls was achieved using 5 mM ammonium acetate in water, 5 mM ammonium acetate in MeOH and ethyl acetate as the mobile phase. According to Gkioni et al. [77], separation of chlorophyll-a and *β*-carotene in samples was problematic using a C18 column. The authors achieved efficient separation and more repeatable results by replacing the acetonitrile with methanol and acetone mixture as the mobile phase.

HPLC elicits a very useful technique for both qualitative and quantitative analysis of carotenoids. The selection of the proper chromatographic column and mobile phase enables the carotenoid analysis of a wide range of samples. Furthermore, the continuous progress of this analytical field, especially in the development of new static phases, can render HPLC even more efficient in carotenoids separation and analysis.

### 4.4. Spectroscopy

Liquid chromatography confers the obvious choice for carotenoids separation and analysis; still, the combination with spectrophotometric methods provides a very useful tool for identification purposes. The most commonly used spectrophotometric methods for carotenoid analysis are mass spectrometry (MS), spectrophotometric analysis with infrared radiation and Fourier transformation (FTIR), Raman spectroscopy and nuclear magnetic resonance (NMR).

Analysis with mass spectrometry (MS) is based on the separation of the different fragments (ions) of carotenoids molecules after their formation from an ionization source. The different molecular fragments have different mass-to-charge ratios (*m*/*z*) and are separated by moving in an electric or magnetic field. MS provides a unique spectrum of the analyte that can be used to identify different compounds even if they have a similar UV–Vis spectrum. The ionization source constitutes an important parameter of the MS analysis, and atmospheric pressure chemical ionization (APCI) and electrospray ionization (ESI) are the most widely used by researchers [46,50,72,76,107,108,109]. For carotenoid analysis, LC-MS has been proved as a very effective and precise technique. Table 2 presents the main parameters affecting the analysis of carotenoids with LC-MS in recently published works. 

On the other hand, analysis with FTIR or NMR provides significant information on the chemical structure and bonds of the analyte. Such analysis can find applications in the identification of the metabolites of already known or newly isolated microorganisms. A typical example is the research of Gurkok [110] regarding the production and identification of a pigment from the strain *Metabacillus idriensis* LipT27. In that work, a combination of TLC, NMR and FTIR analysis was performed in order to identify the newly isolated pigment. After the separation of the pigment with TLC analysis, the formed bands were collected for further analysis with NMR and FTIR. The analysis revealed that the pigment had a carotenoid structure not identical to any other known carotenoid. 

Unlike FTIR, which measures the absorption of infrared radiation, Raman spectroscopy is based on the scattering of incident radiation. The method is based on the fact that specific molecules in a sample scatter most of the incident light at the same wavelength (Rayleigh scattering), while a very small amount is shifted in higher or lower wavelengths (Raman scattering). This phenomenon generates a unique spectrum of the analyte and can be used for its identification. Raman spectroscopy is a non-destructive method of analysis that is increasingly being used for carotenoids. A combination of Raman spectroscopy and HPLC has been reported for the determination of carotenoids in snow algae [111]. In their work, Osterrothová et al. [111] investigated the production of pigments from the green algal species *Chlainomonas* sp., *Chlamydomonas nivalis*, *Chloromonas* cf. *nivalis*, *Scotiella cryophila* and *Chloromonas* sp. In particular, violaxanthin, neoxanthin, antheraxanthin, lutein, *β*-carotene, 13Z-astaxanthin and all-transastaxanthin were determined in green algal samples by means of HPLC analysis. Analysis with Raman spectroscopy of the samples, at different life stages of algae cells, revealed changes in C=C bond stretching frequency due to the different pigment ratios, with the older cells containing more astaxanthin and the younger cells more lutein. 

## 5. Conclusions

Νatural carotenoids demonstrate a plethora of advantages for human health, οwing tο their strοng antiοxidant and anti-inflammatory effects. These traits render carotenoids as compounds of scientific interest, and their biotechnological production seems to be the key for their cost-effective and sustainable production. As in many biotechnological products, recovery and chemical analysis are essential, considering the interrelation with the end applications. Modern extraction techniques ensure “greener” and safer processes to extract carotenoids, whereas parameters optimization is still an ongoing research. Current analytical tools provide an in-depth analysis that could also reveal new compounds and potential carotenoid producers. State-of-the-art research carried out indicates that each microbial strain capable of producing carotenoids necessitates different handling, and thus advantages and limitations of methods should be carefully and meticulously considered.

## Figures and Tables

**Table 2:** Methods for extraction and analysis of carotenoids reported in the literature.

Microorganism	Method of Extraction	Carotenoids	Method of Analysis	Ref
*Xanthophyllomyces dendrorhous*	Cell disruption with zirconia beads and extraction with acetone	Astaxanthin	HPLC—UV detector. Solvent acetonitrile: methanol: 2-propanol (85:10:5) (isocratic)	[51]
*Rhodosporidium kratochvilovae* Y-42 and Y4-3	Cell disruption with DMSO, followed by acetone and solvent extraction with petroleum ether	*β*-carotene, lycopene	HPLC—PDA detector. Solvent: acetonitrile: methanol: THF ^4^ (stabilized with 0.025% BHT) (40:56:4) (isocratic)	[28]
*Chlorella saccharophila*	Solvent extraction with IL (tetrabutyl phosphonium hydroxide)	Lutein	HPLC—UV detector. Solvent A: methanol, solvent B: 200 mM acetic acid and solvent C: MTBE ^1^ (gradient)	[65]
*Synechococcus* sp. PCC7002, *Synechocystis* sp. PCC6803 and *Rhodosorus* sp.	Cell disruption with zirconia beads and extraction with methanol	Zeaxanthin	HPLC—PDA detector. Solvent: methanol: MTBE: water 75:22:3 (isocratic)	[53]
*Pseudomonas* sp. 102515 and genetically modified strains of *E. coli* and *Pseudomonas putida*	Methanol and sonication for the extraction, redissolved in DMSO ^2^—methanol for the analysis	Zeaxanthin diglucoside	HPLC—MS (ESI-MS). Solvent: acetonitrile: water from 50% to 90% (gradient) and methanol: tetrahydrofuran (6:4) (isocratic)	[72]
*Xanthophyllomyces dendrorhous*	DMSO, addition of Na_3_PO_4_ and hexane/ethyl acetate 1:1 (*v*/*v*); redissolved in MTBE	Astaxanthin and *β*-carotene	HPLC—LC/MS. Solvent A: water with formic acid 0.01%/ammonium formate 5 mM; solvent B: acetonitrile—methanol (7:3) methanol with formic acid 0.01%/ammonium formate 5 mM (gradient)	[46,50]
Genetically engineered strains of *E. coli*	Extraction with acetone	Astaxanthin	HPLC—UV detector. Solvent A methanol: acetonitrile: DCM ^3^, 21:21:8 and solvent B: methanol: water, 1:9 (gradient)	[73]
*Chlorella zofingiensis* (mutant)	Extraction with acetone	Zeaxanthin, lutein and *β*-carotene	HPLC—DAD. Solvent A: methanol and solvent B: MTBE (gradient)	[74]
*Sporobolomyces ruberrimus*	Cell disruption with glass beads; extraction with different combinations of hexane, petroleum ether, ethyl ether and acetone	*β*-carotene	TLC analysis with silica gel and acetone: hexane (3:7 *v*/*v*) HPLC—DAD. Solvent A: acetone 99.8% and solvent B: water (gradient)	[70]
*Chlorella sorokiniana*	Extraction with CO_2_-based alkyl carbamate ILs (dipropylammonium dipropylcarbamate, diallylammonium diallylcarbamate, dibutylammonium dibutylcarbamate)	Torulene and torularhodin	HPLC—PDA detector. Solvent: methanol: water 97:3 (isocratic)	[64]
Genetically engineered *Saccharomyces cerevisiae*	Sequential boiling and cooling with 1 N HCl for cell disruption; extraction with acetone	Lutein	HPLC—PDA detector. Solvent methanol: DCM: acetonitrile (47:18:35) (isocratic)	[75]
*Dunaliella salina rubeus* *D. salina salina* *D. salina bardawil*	Extraction with MTBE–MeOH (20:80) assisted by sonication	*α*-carotene, *β*-carotene, lutein and zeaxanthin	HPLC—DAD. Solvent: 80% methanol: 20% MTBE (isocratic)	[49]
*Spirulina platensis*	Supercritical CO_2_ extractions (300 bar and 45 °C)	Zeaxanthin *β*-cryptoxanthin *β*-carotene	UPLC—MS (ESI) analysis Mobile phase acetonitrile: methanol (70:30) (isocratic)	[76]
*Haloarcula* sp. *Halorubrum tebenquichense*	Extraction with acetone: water (8:2) assisted by vortex, sonication and centrifugation	Bacterioruberin	UHPLC—MS analysis Mobile phase solvent A: 1% formic acid aqueous solution, solvent B: methanol with 1% formic acid and solvent C: acetonitrile with 1% formic acid (gradient system)	[42]
*Nannochloropsis oculata*	UAE combined with ES (ethanol of betaine: 1,2 propanediol at a molar ratio of 2:5)	Violaxanthin	LS-MS with mobile phase 0.2% formic acid in water (solvent A), 0.2% formic acid in acetonitrile (solvent B) (gradient system) HPLC—DAD with mobile phase 0.1% formic acid (solvent A), methanol (solvent B), acetonitrile (solvent C), methanol (solvent D) (gradient system) ^1^H-NMR	[77]

^1^ MTBE: methyl tert-butyl ether. ^2^ DMSO: dimethyl sulfoxide. ^3^ DCM: dichloromethane. ^4^ THF: tetrahydrofuran.

## Data Availability

Data is contained within the article.
